# Editorial

**Published:** 1871-04

**Authors:** 


### Editorial.

### *The Uses of a Devil.*

It has always been a puzzling question to persons of a speculative, theological turn of mind, why an All-wise and Benevolent Supreme Being should have permitted the existence of evil in itself, or of the great spirit of evil—the Devil, who is about universally believed to be its prime instigator. It would be so easy to be good, if there were no Devil to tempt otherwise!

But profounder reflection convinces that, for the right and perfect development of human nature, the evil principle is indispensable. Negative goodness has no virtue in it respectable above the positively bad.

Milton, in the *Areopagitica*, puts it thus : "I can not praise a fugitive and cloistered virtue unexercised and unbreathed, that never sallies out and sees her adversary but slinks out of the race, where that immortal virtue is to be run for not without dust and heat. * * * That virtue which is but a youngling in the contemplation of evil, and knows not the utmost that vice promises to her followers, and rejects it, is but a blank virtue, not a pure; her whiteness is but an excremental whiteness."

Hence, in the grand scheme of Providence, a Devil has an essential part, and has his uses. Adam and Eve before "the fall" must have been little above Huxley's Protoplasm in deserving. The Devil brought the fall, that brought both toil—"Brain-sweat and Brow-sweat"—and that mental and moral development which lifts man to his real place, "a little lower than the angels," but a good way above the Devil. "There is a soul of goodness in things evil." "Partial ill is universal good." There is an element of truth even in a lie, for it suggests its opposite; the lighthouse shows what to avoid, as the old time "Pillar of Fire" showed the path to be followed.

Which train of reflection leads us directly to medicine, formerly a part of theology practically. Indeed, even now, when divorced, (like the severed halves, male and female, of ancient philosophy, that went wandering up and down the earth seeking their mates, *sometimes* finding the right ones, but, alas, too often the wrong ones,) medicine and theology have striking affinities—Doctors are polemical, and priests dabble in physic. Medicine, like theology, has its evil principle, or, rather, its "Legion." Without these, it is questionable whether its highest development or perfection could be secured. Bacon called them "Idols"—effigies, images, representations, emblems, of evil spirits, "hot from Tartarus." Let us call them the *Legion* not yet wholly cast out from the collective medicine-man. Some of them are:

Bald Empiricism, which believes in the *post hoc, ergo propter hoc*, and catalogues its remedies and its cures. *Figurative* truths, established not by the Tables of the *Law*, but by the multiplication table.

Sheer Dogmatism, which assumes that the truths of life, health, disease and remedy can be cabinned, cribbed and confined in some wretched catchwords like Hahnemann's sibilant syllables.

False Philosophy, that fastens upon half a dozen palpable facts (misinterpreted), a hundred more assumed (but baseless), and then babbles of simplicity and natural law, as if the grand secrets of nature had already been distilled from its pitiful alembics, or dug out by its dull scalpels. The experimental chemists and physiologists give us enough of this, not to speak of the microscopists and metaphysicians. The hewers of wood and drawers of water for the science of medicine, have each of them their "ready method" with the universe.

Rank Absurdity, that receives *omne ignotum pro magnifico*, that revels in marvelous cures and magical appliances; puts buckeyes in the breeches pockets to cure piles; makes "passes" for neuralgia; titillates megrims, hyp. and hysteria with pellets and potencies; or rises to the level of the sublime by grand "perturbations," with "heroic" doses of calomel, opium, veratrum, *et id omne genus;* or goes to the depth of bathos with wet sheets and ice water, or blood-letting and hot water, with Death himself checking "retrograde metamorphosis" and facilitating "elimination" by his co-operative fingers.

A few specimens only of the Legion, yet a catalogue too grim to laugh at, too ridiculous to grow lugubrious over.

We confess to the firmest faith and strongest confidence in medicine, both as a science and an art, nevertheless the most painful conviction is forced upon us, that the greatest obstacle to its progress is found, not so much in the operations and attacks of the *outside* medical sectaries, as in the constant neglect of so large a proportion *within* the profession, of the primary and fundamental teachings which they claim to accept.

The best of us are, too often, the dupes of philosophical inconsistency. From Huxley, with his splendid vaporings, down to the humblest correspondent of the medical press, there is all the time a manifest anxiety to escape, in the discussion of medical topics, the trammels of cool scientific method, and to substitute the scintillations of genius and imagination for the solid deductions of close observation and logical thought. The medical sectaries and the specialists, each with their little bundle of truths and vast piles of fiction, illustrate the uses of the Devil, and there is no advantage in fretting or fuming over their absurdities. Some show what nature can do unassisted, and others how much the human system can endure and yet escape with life.

Travelers in the tropics learn from the monkeys what unknown fruits they may eat with impunity, and the cautious stranger watches the bibulous customers of an Indiana bar, before he ventures upon the "sudden-death" or "forty-rod" dispensed from its decanters.

Each new method, and new medicine, may be judiciously left to the wholesale use of its enthusiastic advocates, whilst thoughtful physicians from its "morbid anatomy" may gather clearer ideas. Let the believers in specifics indulge in their most dangerous experimentation, they will lessen the necessity for Malthusian methods of keeping down the excess of population.

The search for the Philosopher's Stone and the Elixir of Life, brought to light abundance of useful truth.

The rise, progress, culmination, decline and fall of individual methods and "systems" may entitle medicine to be called a "History of Variations," as Sir William Hamilton objected, but even in its variations it has "a philosophy teaching by examples," whereby profit will accrue to the judicious thinker.

In theology, the day of marvels and miracles, nay even of

"Special Providences," has confessedly passed—although the spirit of evil is still unchained.

In medicine, there is analogous change, but the millenium has not yet dawned—its devil of False Philosophy is still loose.

***An Organ-ic Error—***

That this *Journal* is the "organ" of Rush Med. College, or of any other college, society, clique or individual. We ask our correspondent on another page, to take notice. The *Journal* is the "organ" of the medical profession as a whole. All opinions can be freely expressed on its pages, provided those who entertain them do so intelligently, and cheerfully submit them to the wholesome criticism which Medicine in its present high stage of enlightenment everywhere demands. We repeat, what we have elsewhere written, we insist upon *good faith.* When we believe this to be present, we shall admit a well written article though we differ, personally, *toto cœlo* from the conclusions of its author.

The present writer is, perhaps from long experience, somewhat unusually pachydermatous, nevertheless he never could understand why some people are so particularly sensitive when their pet notions are attacked. They ought rather to rejoice at the opportunity afforded to show their tenets sound, their positions invincible, or else to discard the former and scramble down from the latter. Beware of thin skins, Dear Brethren.

It is a pitiful pride which plumes itself on holding precisely the same views now as twenty years, or one year, or even a day ago. Are we to learn nothing, to achieve nothing, to throw away nothing, or forget nothing ? Better be put away in salt and carbolic acid pickle at once. The Icthyosaurus is a good type of stability, but it is time enough to fossilize when dead.

***Carrion Flies.***

The severe *critique* which appeared in the *Journal* upon a recently published obscene book, (the naked villainy of which was even more apparent from the odd ends stolen from holy writ, that, here and there, bedizened it,) has awakened the intense wrath of the noisome insects who sat down to batten on its reeking rottenness. They fill their little circles of foul air with epithets and threatenings. Meanwhile every respectable Medical Journal in the country, which has noticed it at all, has denounced it in terms to the full as energetic as those used by "*Volta Reeke*" or the Editor himself. That old and respectable paper, the *Boston Medical and Surgical Journal*, refused even to mention the name of the book in its pages, but after quoting from our correspondent and endorsing his views completely, says: "Our brother editor, in his remarks, truly stigmatizes the book as 'the culminating atrocity of the press.' Having made these remarks, *our* copy of the book goes to the flames." And yet prurient parsons in this city, to our knowledge, have put copies of the book into the hands of pure minded ladies, and howl, like baffled wolves, because we have exposed its disgusting enormities. We denounce the clergyman who does this from this time forth as a moral leper, and advise the lady to whom it is recommended, to imitate the editor of the *Boston Journal*, and consign the book to the flames, and the presentor to—Coventry, or a warmer climate.

### *Bliss & Sharp — Removal.*

The well-known firm, Bliss & Sharp, recently of 144 Lake St., have removed to the spacious and elegant store, 105 State St., in the magnificent block adjoining "Booksellers' Row," wherein our Publishers find lodgement for their immense stock of literary and stationery wares. Calling upon them a few days since we found ourselves surrounded by everything needed or desired by the most devoted admirer of drugs, and every convenience for dispensing, sale or show of the Materia Medica, together with all those æsthetic appurtenances which our apothecaries know well how to dispose so as to attract the eye and gratify the sense. Those in the habit of visiting their old but popular store, will be pleased to learn that the department for surgical instruments, and apparatus of all sorts, has been assigned a distinct room on the second floor, reached by a broad and easy stairway from the central portion of the main apartment. In this department can be found about everything, in the way of mechanical appliances, the Physician, Surgeon, Obstetrician or Specialist of any variety may need.

Those who may not wish to purchase will nevertheless be gainers by looking over the immense variety here presented. They will see, in a *coup d'œil*, the wonderful progress which has been made, within a comparatively recent period, in the creation of material aids to the profession. We advise our readers to look over this magazine, and thus secure many valuable hints for the future.

The same enterprising firm have also removed their branch store, 125, 22d St., to the beautiful building on the corner of the same street and Wabash Avenue. The branch store, although by no means as large as the palatial "down town" place, is, nevertheless, a *bijou* in its way, and did space permit would have a more extended notice. It is unnecessary for us to say that with Messrs. Bliss & Sharp purity and perfection of medicines is a cardinal and ever present virtue. As a class, Chicago may well feel pride in many of its apothecaries, and point to this house as especially distinguished.

***Thanks to Contemporaries***

Are due and cheerfully tendered for copies of missing back numbers of their respective journals. We shall always be happy to reciprocâte to any extent in our power. We prize the Medical Journals of the time very highly, and not only file them, but have them bound, and their important articles minuted in a general Library Index.

***Back Numbers Wanted.***

To complete files, Twenty-five cents each will be paid for the following Nos. of the JOURNAL for 1869: June, 1, No. 11; July 15, No. 14; August 1, No. 15; August 15, No. 16.

*To the Editor of the Chicago Medical Journal:*

Cook County Hospital was lately the scene of a pleasant gathering, the occasion being the annual reunion of Interns. Four years have passed since the first certificate of the Medical Board was granted. Dr. N. T. Quales, the present Superintendent of the United States Marine Hospital in Chicago, was the recipient. Others have been added to the list of foster children at the rate of three each year. The young men so distinguished have done credit to their alma mater. Some of them have already achieved a fair position in the practice they have chosen. They all cherish the memories of an arduous service, and love with apostolic fervor their companionships therein. They will strive to ennoble the career of the Hospital.

It was for the purpose of perpetuating friendship and self-improvement that the Association was formed one year ago, Dr. Quales acting as the first President, and Dr. T. M. Hutchinson, Secretary. The reunion this year was a success, and will be long remembered. A bountiful supper was provided by the Warden, of whose hospitality all had tasted before. Other exercises of a scientific interest followed. At a late hour the assembly dispersed, to meet again in one year.

C. T. Fenn.

March 20, 1871.

---

### *Alumni of the Med. Dept. of the University of the City of New York.*

The Executive Committee of the Alumni Association of the Medical Department of the University of the City of New York purpose the publication, at the earliest possible date, of a complete catalogue of the graduates from that institution since its foundation. The records of the Faculty having been destroyed in the burning of the college building some years ago, this project is one that should be seconded by every one of the alumni, of whom between two and three thousand are scattered throughout the United States. It is earnestly requested that each of these will, without delay, forward for enrolment his full name and post office address, with his professional history, including date of graduation, posts of honor and trust held, etc., and also any information which he may possess concerning former class-mates who have since died or retired from practice. Communications should be addressed to the Secretary, Chas. Inslee Pardee, M.D., 72 West 35th street, New York.

